# Bradykinin type 2 receptor -9/-9 genotype is associated with triceps brachii muscle hypertrophy following strength training in young healthy men

**DOI:** 10.1186/1471-2474-13-217

**Published:** 2012-11-06

**Authors:** Jelena Z Popadic Gacesa, Milica Momcilovic, Igor Veselinovic, David A Brodie, Nikola G Grujic

**Affiliations:** 1 Laboratory for Functional Diagnostics, Department of Physiology, Medical School, University of Novi Sad, Novi Sad, 21000, Serbia; 2 Centre for Molecular Genetics, Institute for Legal Medicine, Clinical Center of Vojvodina, Novi Sad, 21000, Serbia; 3 Medical School, University of Novi Sad, Novi Sad, 21000, Serbia; 4 Faculty for Society and Health, Buckinghamshire New University, Uxbridge, UK

**Keywords:** Bradykinin, Muscle hypertrophy, Strength training

## Abstract

**Background:**

Bradykinin type 2 receptor (*B2BRK)* genotype was reported to be associated with changes in the left-ventricular mass as a response to aerobic training, as well as in the regulation of the skeletal muscle performance in both athletes and non-athletes. However, there are no reports on the effect of *B2BRK* 9-bp polymorphism on the response of the skeletal muscle to strength training, and our aim was to determine the relationship between the *B2BRK* SNP and triceps brachii functional and morphological adaptation to programmed physical activity in young adults.

**Methods:**

In this 6-week pretest-posttest exercise intervention study, twenty nine healthy young men (21.5 ± 2.7 y, BMI 24.2 ± 3.5 kg/m^2^) were put on a 6-week exercise protocol using an isoacceleration dynamometer (5 times a week, 5 daily sets with 10 maximal elbow extensions, 1 minute rest between sets). Triceps brachii muscle volumes were assessed by using magnetic resonance imaging before and after the strength training. Bradykinin type 2 receptor 9 base pair polymorphism was determined for all participants.

**Results:**

Following the elbow extensors training, an average increase in the volume of both triceps brachii was 5.4 ± 3.4% (from 929.5 ± 146.8 cm^3^ pre-training to 977.6 ± 140.9 cm^3^ after training, p<0.001). Triceps brachii volume increase was significantly larger in individuals homozygous for −*9* allele compared to individuals with one or two +*9* alleles (−*9*/-*9*, 8.5 ± 3.8%; vs. -*9*/+*9* and +*9*/+*9* combined, 4.7 ± 4.5%, p < 0.05). Mean increases in endurance strength in response to training were 48.4 ± 20.2%, but the increases were not dependent on *B2BRK* genotype (−*9*/-*9*, 50.2 ± 19.2%; vs. -*9*/+*9* and +*9*/+*9* combined, 46.8 ± 20.7%, p > 0.05).

**Conclusions:**

We found that muscle morphological response to targeted training – hypertrophy – is related to polymorphisms of *B2BRK*. However, no significant influence of different *B2BRK* genotypes on functional muscle properties after strength training in young healthy non athletes was found. This finding could be relevant, not only in predicting individual muscle adaptation capacity to training or sarcopenia related to aging and inactivity, but also in determining new therapeutic strategies targeting genetic control of muscle function, especially for neuromuscular disorders that are characterized by progressive adverse changes in muscle quality, mass, strength and force production (e.g., muscular dystrophy, multiple sclerosis, Parkinson’s disease).

## Background

Skeletal muscle adaptation to programmed physical activity includes changes in muscle function and morphological properties [1,2]. The extent of this specific adaptation is influenced by multiple factors, such as genetic predisposition, hormonal influences, the neural component, muscle fiber composition, characteristics of training (frequency, intensity and duration), and others [1,3]. A number of genes have been discovered that are likely to influence specific performance both in athletes and non-athletes. In the decade after the gene for angiotensin converting enzyme (*ACE*) was first proposed to be a ‘human gene for physical performance’ [4,5], there have been numerous studies examining the effects of *ACE* and other genes on athletic status [6,7]. In several cases, these gene variants have been associated with aerobic capacity and muscle fiber composition as endurance related traits [6,7].

Bradykinin is a nonapeptide that acts as a potent vasodilator [8]. Within skeletal muscle, bradykinin promotes glucose uptake and alters muscle blood flow [9-12]. Bradykinin exerts many of its effects by binding to bradykinin type 2 receptor (*B2BRK*) [13]. Transcriptional gene activity and mRNA levels of *B2BRK* are influenced by the nine base pair (9-bp) repeat fragment in exon 1. Lower transcriptional activity and lower mRNA levels are detected in the presence of the 9-bp fragment (+9), while the absence of the 9-bp fragment (−9) leads to higher transcriptional activity and mRNA levels [14,15].

Several studies have implicated *B2BRK* in the adaptation to exercise and in the regulation of the skeletal muscle performance. Response to a 10-week training programme resulted in changes in the left-ventricular mass that were greatest for the carriers of the +*9* allele, and smallest for the −*9* homozygotes [16]. Bradykinin −*9* allele was also associated with greater skeletal muscle metabolic efficiency in both athletes and non-athletes [17]. However, there are no reports on the effect of *B2BRK* 9-bp polymorphism on the response of the skeletal muscle to strength training. We have recently reported the morphological changes of triceps brachii muscle as a result of 6 and 12-week strength training protocols, and the correlation of muscle volume with changes in functional muscle properties [18,19]. Although a correlation was discovered between morphological and functional properties of muscles, there was no significant correlation between changes in muscle volume and strength as a result of the resistance training. Also, participants showed different range in strength and volume increase after maximal training stimulus applied, which additionally implied the possible influence of genetic predisposition to muscle adaptation capacity.

In accordance with these data and previous studies that had found association between bradykinin type 2 receptor (*B2BRK*) and muscle hypertrophy, we followed triceps brachii morphological and functional changes as a result of strength training. We chose to train elbow extensors, since we could isolate extension movement and precisely measure the strength of each contraction. Also, these are not postural muscles and therefore we could control training stimulus more closely then with other muscles (for example, leg muscles). Since apparent hypertrophy occurs after first four weeks of training, our aim was to follow-up this initial hypertrophic response to the training stimulus.

The aim of this study was to determine the influence of bradykinin type 2 receptor 9-bp polymorphism on the response of triceps brachii adaptation to a 6-week training of elbow extensors.

## Methods

### Subjects

Twenty nine healthy young Caucasian men (21.5 ± 2.7 y, body mass (BM) 80.2 ± 12.6 kg, body height (BH) 181.7 ± 7.2 cm, body mass index (BMI) 24.2 ± 3.5 kg/m^2^), who did not take part in any formal resistance exercise regime for 6 months prior to starting a 6-week exercise protocol, volunteered for this study. They were all healthy, according to the anamnestic data. All subjects were given an oral and written explanation of the study before signing a written informed consent form.

The study was approved by the Research Ethics Committee of the Medical School, University of Novi Sad, and the investigation was performed according to the principles outlined in the Declaration of Helsinki.

### Training protocol and triceps brachii muscle strength measurements

Elbow extensors training was performed on the isoacceleration dynamometer Concept 2 Dyno (Concept 2, Inc. Morrisville, Vermont, USA). The testing–training procedure had been introduced to the participants one day prior to the training in order to make them familiar with the procedure. The training protocol lasted for 6 weeks, with a frequency of five sessions per week (5 consecutive training days during a working week, and two days without training during the weekend – this protocol was the same for all participants). Each training session included five sets of 10 maximal elbow extensions following 15 minutes of a general warm-up. A one minute resting period was allowed between each set. Each contraction was performed in the sitting bench press position with the full elbow extension of both arms against the resistance to maintain a central acceleration during the whole range of movement, as previously reported [18,19]. All participants were regularly attending their sessions throughout the whole training.

Adequate and precise movement range was accomplished with the constant supervision of training series by the investigator. We instructed participants how to perform contractions, controlled all the movements during the training and we asked them to perform all extensions correctly and with maximal strength. We were encouraging them verbally during the entire process. They were always measured in a group so they could observe training sessions preceding their own, which potentiated a competitive spirit among them.

The isoacceleration dynamometer uses constant acceleration throughout the whole range of movement – the measured load and muscle strength represent the average value achieved during the whole movement duration. Therefore, the isoacceleration dynamometer which was used in this study allowed a precise measurement of each contraction workload and strength output. The participants were performing contractions with strength which they personally perceived to be their maximal strength, and load and strength were measured for every single contraction, which enabled us to exactly measure individual and total workload and strength during a series of contractions. Therefore, the load was individually applied and maximal in each contraction, according to the personal perception of each participant. The training load was not dosed; it was the result of the participants’ maximal muscle strength – therefore individually adjusted.

Strength of each elbow extension was recorded during the training sessions and used in the subsequent analysis. Maximal strength (MS) was defined as the highest scored result in Newtons (N), while endurance muscle strength (ES) of both triceps brachii muscles was defined as the average value of all 50 contractions expressed in Newtons (N). Percentage decline of average values of the fifth series in relation to the first one was defined as fatigue rate (FR). All three strength parameters were measured on the first and the last (30^th^) day of training.

### Muscle volume measurements

**Figure 1:**
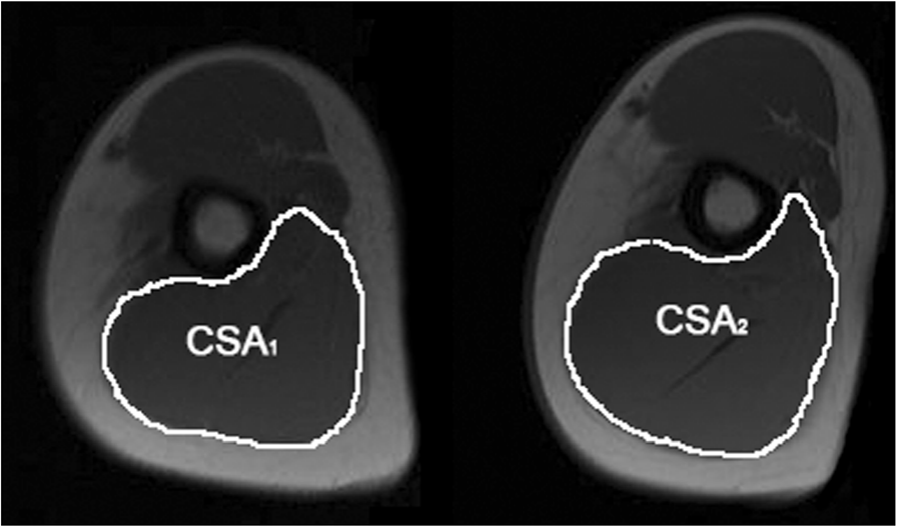
**Two images of a slice at mid**-**humerus in a participant before and after 6**-**week strength training.** The manually outlined cross-sectional areas (CSA) of m.triceps brachii show significant increase in muscle area – hypertrophy as a result of elbow extensors training (before training CSA_1_ = 27.3 cm^2^, and after training CSA_2_ = 29.5 cm^2^).

A series of cross-sectional images of the triceps brachii muscles were obtained by MRI scans with an extremity coil on the Magnetom Avanto TIM Siemens, 1.5 T (Siemens, Erlangen, Germany). Мultislice sequences of T1-weighted, flash (gradient echo) axial images of both arms were obtained, starting from the humerus head to the medial epicondils, and each arm was imaged separately. MRI parameters were: repetition time (TR) – 232 ms, echo time (TE) – 4.76 ms, field of view (FOV) – 162 × 288 mm, and the matrix size – 288 × 512. Slice thickness was 10 mm with an interslice interval of 3 mm. Data processing was conducted using the Medical Image Processing, Analysis and Visualization (MIPAV) software, version 2.6 (Center for Information Technology, National Institutes of Health, Bethesda, MD). The MIPAV software was used to analyze images on a personal computer workstation [20]. This program calculates the surface area (in mm^2^) of the manually selected region of interest – ROI (i.e. triceps brachii cross-sectional area – CSA) in every axial image from the first section closest to the superior border of the humerus to a point where the muscle group is no longer reliably distinguishable [21,22]. Two post-processed images of a single slice at mid-humerus in a participant, before and after-training with outlined m.triceps brachii CSAs, are shown in Figure 1. The same number of sections distal from the humerus was measured for a particular subject before and after 6 weeks of training, to ensure identical measurement replication. All sequences were analyzed by the same person (JPG). The investigator was blinded to subject identification, date of scan, and training status, as well as their genotypes, in both baseline and after training analyses. The intra-class correlation coefficient for repeated measurements was 0.99. Cronbach’s Alpha (α) - Reliability Coefficient for repeated measures on the same participant was 0.98 for triceps brachii. The final muscle volume (V_m_) was calculated using the truncated cone formula, as previously reported [23]. Final muscle volume used for further calculations was the sum of both arms triceps brachii muscle volumes. For all participants, final volume measurements on MRI were performed three days after the last training session, with the same protocol applied.

### Genotyping

Genomic DNA was isolated from blood collected on FTA cards (Whatman) using Chelex [24]. In order to identify 9-bp insertion/deletion polymorphism within the bradykinin type 2 receptor gene PCR reaction was performed according to a previously reported protocol [25]. Briefly, forward (5' – TCCAGCTCTGGCTTCTGG –3') and reverse (5'– AGTCGCTCCCTGGTACTGC –3') primers were combined with 1x AmpliTaq Gold buffer (Applied Biosystems), 3.75 mM MgCl_2_, 800 uM dNTPs (Fermentas), 1 U AmpliTaq Gold polymerase (Applied Biosystems) and genomic DNA. PCR conditions included initial denaturation at 94°C, followed by 40 cycles of denaturation at 94°C, annealing at 60°C and elongation at 72°C followed by a final elongation step at 72°C. PCR products were resolved on 3% agarose gels.

### Statistical analysis

All data were presented as mean ± SD unless otherwise indicated. Hardy-Weinberg equilibrium was tested using a Chi-square analysis with one degree of freedom. For the statistical analysis triceps brachii volumes were calculated for each arm, and then combined. The differences between *B2BRK* genotypes were determined using One-way Analysis of Variance (ANOVA) or Student’s two-tailed *t* test when appropriate (for comparisons between −*9* homozygotes and two other genotypes combined). When indicated by a significant *F* value, Tukey’s test was performed to identify differences between genotype groups. Statistical significance was indicated if p < 0.05. Minimum required sample size for our study was 5 for both groups, for the following parameters: number of groups = 2, type I error rate α = 0.05, statistical power 0.8, average value (standard deviation): for sample one AVE = 68.9 (24.3) cm^3^; for sample two AVE = 49.4 (46.4) cm^3^. All calculations were performed using the Statistica data analysis software (StatSoft).

## Results

*B2BRK* genotype distribution (n = 6 -*9*/-*9*, n = 18 -*9*/+*9*, n = 5 +*9*/+*9*) was consistent with Hardy-Weinberg equilibrium. Allele frequencies were 0.52 for −*9* allele and 0.48 for +*9* allele, which is similar to those previously reported [14-17]. Pre-training characteristics (age, weight, height, body mass index) were independent of *B2BRK* genotype.

### Muscle strength changes

**Figure 2:**
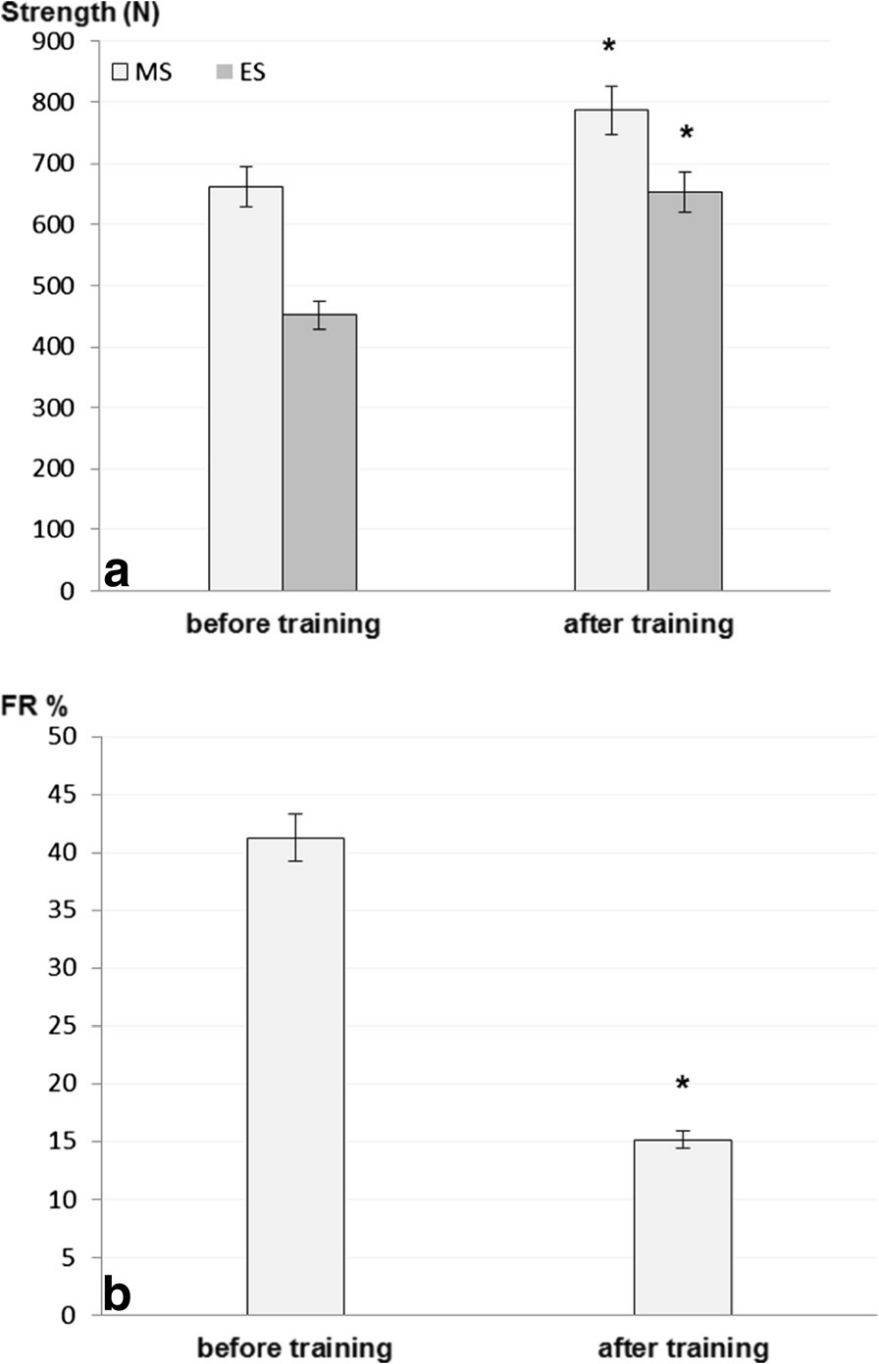
**Values of two functional parameters in all participants(N= 29)expressed before and after the training: a)maximal and endurance strength values in Newtons**, **and b)fatigue rate as a percentage of strength decrease.** Maximal and endurance strength showed significantly higher post-training values, while fatigue rate decreased significantly after strength training of elbow extensors. However, no significant changes in these parameters were observed in association with *B2BRK* genotypes. Legend: MS – maximal strength; ES – endurance strength; FR – fatigue rate; N – Newtons. * p < 0.05.

*B2BRK* genotype did not have any influence on pre-training maximal or endurance triceps brachii muscle strength (MS for −9/-9: 623.5 ± 126.5 N vs. -9/+9 and +9/+9: 671.6 ± 120.6 N; p = 0.19; ES for −9/-9: 426.5 ± 83.3 N vs. -9/+9 and +9/+9: 456.9 ± 108.8 N; p = 0.21) or post-training triceps brachii strength (MS for −*9*/-*9*: 753.9 ± 126.5 N vs. -*9*/+*9* and +*9*/+*9*: 795.1 ± 119.6 N; p = 0.23; ES for −*9*/-*9*: 635.3 ± 117.6 N vs. -9/+9 and +*9*/+*9*: 657.8 ± 120.6 N; p = 0.31). Similarly, fatigue rate showed no significant correlation with *B2BRK* genotype (pre-training −*9*/-*9*: 41.2 ± 13.4% vs. -*9*/+*9* and +*9*/+*9*: 41.3 ± 10.2%; p = 0.49; post-training −*9*/-*9*: 15.8 ± 6.6% vs. -*9*/+*9* and +*9*/+*9*: 14.9 ± 7.4%; p = 0.38). Mean increases in functional muscle properties in response to 6 weeks of training were significant (for MS 19.9 ± 11.4%, for ES 48.4 ± 20.2%, and decrease in FR 73.8 ± 10.3%), as presented in Figure 2a and 2b. However, these changes were not dependent on *B2BRK* genotype (p > 0.05).

### Muscle volume changes

To determine if changes in muscle volume in response to training were dependent on *B2BRK* genotype, we recorded the MRI of the upper arm before and after 6 weeks of training and examined the influence of *B2BRK* genotype on muscle gains.

Pre-training triceps brachii volume was not dependent on *B2BRK* genotype: -*9*/-*9*, 845.7 ± 137.3 cm^3^; vs. -*9*/+*9* and +*9*/+*9* combined, 950.6 ± 148.7 cm^3^ (p = 0.17). Bradykinin polymorphism did not influence post-training triceps brachii volumes, as well: -*9*/-*9*, 914.2 ± 124.5 cm^3^; vs. -*9*/+*9* and +*9*/+*9* combined, 993.4 ± 147.2 cm^3^ (p = 0.28).

After 6 weeks of elbow extensors training, an average increase in the volume of triceps brachii muscle was 5.4 ± 3.4% as pre-training volume increased from 929.5 ± 146.8 cm^3^ to 977.6 ± 140.9 cm^3^ after training (p <0.001).

Bradykinin receptor genotype did have an effect on the extent of the change in triceps brachii volume, both expressed as difference (ΔV −*9*/-*9*, 68.9 ± 24.3cm^3^; vs. -*9*/+*9* and +*9*/+*9* combined, 49.4 ± 46.4cm^3^ (p = 0.04), and as percentage change (V% -*9*/-*9*, 8.5 ± 3.8%; vs. -*9*/+*9*, and +*9*/+*9* combined, 4.7 ± 4.5% (p = 0.03). Increase in triceps brachii volume was significantly larger in individuals homozygous for −*9* allele compared to individuals with one or two +*9* alleles.

## Discussion

The aim of this study was to determine the possible influence of bradykinin type 2 receptor 9 base pair polymorphism on triceps brachii muscles hypertrophy as a result of six weeks of self-perceived maximal elbow extensors strength training. Since muscle adaptation is in part influenced by heritability, we wanted to investigate the possible influence of bradykinin type 2 receptor gene variants on skeletal muscle adaptation capacity in young adults.

The volume of upper arms elbow extensors increased significantly, by 5.4 ± 3.4%, as a result of a 6-week strength training of our participants. These data are in line with other researchers, since skeletal muscle hypertrophy is a part of muscle adaptation to increased physical activity levels. Different resistance training protocols have been shown to present a variety of exercise demands resulting in adaptations which are specific to the exercise program used [1-3]. Andersen et al. found that m.quadriceps femoris CSA at mid-femur increased by 10% after 3 months resistance training [2]. Abe et al. found relative increases in upper (12% - 21%) and lower (7% - 9%) body muscle thickness in men and 10% - 31% and 7% - 8% in women, respectively [1]. This was after 12 weeks of resistance training, three times per week (six dynamic resistance exercises to fatigue using 8–12 1-repetition maximum – 1RM) in untrained participants [1].

Skeletal muscle adaptation capacity depends on numerous factors, and the genetic predisposition is certainly one of them. There are number of reports in literature regarding influences of different genes and their polymorphism on skeletal muscle morphological and functional adaptation. In our study, we used MRI to evaluate muscle hypertrophy response to training stimulus, which was maximal according to participants’ personal perception, and investigated if there was an influence of bradykinin type 2 receptor variants on the extent of hypertrophy and strength gains.

We found that the presence of two −*9* alleles of the *B2BRK* leads to significantly higher skeletal muscle hypertrophy after a short-term strength training, compared to the presence of one or two +9 alleles. Montgomery’s group reported that heart muscle hypertrophy is more extensive in individuals with +9/+9 genotype variant, when completing a 10-week physical training protocol [5]. They showed that genotypes that lead to lower bradykinin type 2 receptor concentrations (*B2BRK* +9/+9) correlate with greater cardiac growth response to increased physical activity. Our data suggest that skeletal muscle responds differently from cardiomyocytes to the increased load. Although pre-training triceps brachii muscle volume and average strength did not appear to be influenced by the bradykinin 9-bp polymorphism, triceps brachii muscle volume increased significantly more in individuals with −9/-9 genotype after 6 weeks of targeted training.

We discovered a significant change in muscle functional properties as a result of strength training. Maximal muscle strength increased by 19.9%, endurance by 48.4%, and fatigue rate decreased by 73.8%. However, bradykinin polymorphism did not correlate with the changes in triceps brachii muscle strength, endurance and fatigue, as a result of a 6-week training. There are only a few reports in the literature on the relationship between polymorphism in the bradykinin type 2 receptor and the functional capacity of skeletal muscles. Williams et al. reported that common 9-bp variation in the *B2BRK* is associated with efficiency of skeletal muscle contraction (delta efficiency) in both athletes and non-athletes [17]. On the other hand, Hopkinson and associates reported that the genotype with reduced *B2BRK* expression (+9) is associated with quadriceps strength in chronic obstructive pulmonary diseases [26].

A possible explanation of our results concerning muscle strength, which are not in line with some other researchers, may be found in the fact that there is a variety of functional muscle properties that can be assessed as a result of specific adaptation to training, and we followed only some of them. It is known that muscle strength is not completely dependent on muscle size, and there are a number of factors that influence strength, endurance, or fatigue, and their change after training, especially in the first 4–6 weeks of programmed physical activity.

Higher transcriptional activity and higher mRNA levels have been associated with the *B2BRK* −9 allele. Previous work showed that bradykinin could increase glucose uptake in the skeletal muscle during exercise [9,27]. Activation of bradykinin type 2 receptor could also result in an increased concentration of inositol triphosphate, which gives rise to increased calcium concentration within the cell [28,29]. Bradykinin has also been shown to induce the production of nitric oxide in the skeletal muscle [30], which can reduce oxygen consumption in skeletal muscle mitochondria [31]. It can be expected that the higher number of bradykinin type 2 receptors could lead to increased stimulation of skeletal muscle cells when exposed to training stimulus. It is all mediated throughout the increased substrate intake and therefore throughout the increased energy production, which altogether stimulates cell activity and promotes hypertrophic stimulation mechanisms.

Our results suggest that higher bradykinin receptor levels present in individual homozygous for −*9* allele of the *B2BRK* gene may be advantageous in the adaptation of the skeletal muscle to the demands of the training. Ability to gain muscle is especially important in later decades of life, when loss of muscle bulk correlates with poorer quality of life and higher mortality [32]. Identifying gene polymorphisms that influence muscle responsiveness to training could help identify those with higher probability of muscle loss, as well as aid in designing more tailored approaches to training. This finding could be relevant, not only in predicting individual muscle adaptation capacity to training or sarcopenia related to aging and inactivity, but also in determining new therapeutic strategies targeting genetic control of muscle function, especially for neuromuscular disorders that are characterized by progressive adverse changes in muscle quality, mass, strength and force production (e.g., muscular dystrophy, multiple sclerosis, Parkinson’s disease).

Certain limitations need to be considered in the present study. The number of participants was small, especially in a +9/+9 genotype group (only five), and therefore we presented our data set divided into two groups only – one without, and the second with the presence of one or both +9 allele. The reason for this was the fact that the presence of both −9 alleles leads to higher expression of bradykinin receptors, compared to other two genotype combinations. The exercise used in the training could partially engage some other muscles in this area (etc. deltoideus and pectoralis). However, the specific position which the participant had taken (upright seated position, with elbows close to the trunk) and the fact that every session was closely monitored by the investigator, allow us to assume that triceps stimulation was equal during the training process, and that the participation of other muscles was not significant. The aim was not to trace a single trait, its’ distribution in population, nor its’ association with some functional or morphological properties of skeletal muscles. We wanted to explore the possible influence of *B2BRK* polymorphism on skeletal muscle adaptation capacity after a programmed physical activity protocol, and these changes were followed in the same participant using precise measurements. Also, by applying the identical training and testing procedures in all participants, we additionally diminished the possibility that strength values or their increase were overestimated in some volunteers.

## Conclusions

Six week maximal, self-perceived strength training of elbow extensors led to significant adaptation changes of triceps brachii muscles. Elbow extensors of −*9*/-*9 B2BRK* homozygous individuals exhibited significantly higher hypertrophy compared to those with −*9*/+*9* or +*9*/+*9 B2BRK* variants. However, there was no significant influence of different *B2BRK* genotypes on functional muscle properties after strength training in young healthy non athletes.

## Abbreviations

ACE: Angiotensin converting enzyme; B2BRK: Bradykinin type 2 receptor; mRNA: Messenger RNA; BM: Body mass; BH: Body height; BMI: Body mass index; MS: Maximal strength; ES: Endurance muscle strength; FR: Fatigue rate; TR: Repetition time; TE: Echo time; FOV: Field of view; ROI: Region of interest; CSA: Cross-sectional area; V_m_: Muscle volume; 1RM: 1-repetition maximum; AVE: Average value; SD: Standard deviation.

## Competing interests

The authors declare that they have no competing interests.

## Authors’ contributions

JPG designed the study, supervised exercise trainings, collected, analyzed and interpreted data, performed MRI measurements and post-processing, and drafted the manuscript. MM performed genotyping and analyzed data, did statistics, analyzed and interpreted data, and drafted the manuscript. ISV performed genotyping and analyzed data. DAB and NGG designed the study, and revised it critically for important intellectual content. All authors contributed to the final critical revision of the manuscript.

## Pre-publication history

The pre-publication history for this paper can be accessed here:

http://www.biomedcentral.com/1471-2474/13/217/prepub
